# Sex‐Biased and Density‐Dependent Natal Dispersal in a Highly Mobile but Philopatric Raptor

**DOI:** 10.1002/ece3.71487

**Published:** 2025-05-26

**Authors:** Bernadett Zsinka, Szilvia Kövér, Márton Horváth, Nóra Vili, Veronika Szabó‐Csonka, Krisztián Szabó, Szilvia Pásztory‐Kovács

**Affiliations:** ^1^ Department of Zoology University of Veterinary Medicine Budapest Budapest Hungary; ^2^ Lendület Ecosystem Services Research Group Institute of Ecology and Botany, HUN‐REN Centre for Ecological Research Vácrátót Hungary; ^3^ MME BirdLife Hungary Budapest Hungary

**Keywords:** DNA profiling, eastern imperial eagle, local density, sex‐biased dispersal

## Abstract

Natal dispersal plays an important role in the ecological and evolutionary processes of a species and has major implications for its conservation. Here, we studied natal dispersal patterns in the Pannonian population of the vulnerable eastern imperial eagle *Aquila heliaca* in regard to sex, local density, and dispersal direction. We defined local density at each nest as the reciprocal of the area of the Thiessen polygon drawn around the nest. Using the combined methods of colur‐ringing, GPS tracking, and DNA profiling, we managed to determine natal dispersal distances for 116 chicks (43 males, 72 females, 1 with unknown sex) hatched in Hungary between 2012 and 2020. Eagles were highly philopatric, with all 116 individuals settling in the Pannonian population and only 4.3% of them emigrating from the eastern to the western subpopulation. Natal dispersal distance was female‐biased (median: 57.6 km for females and 35.9 km for males). In general, birds settled at lower‐density sites compared to their natal site but moved to higher‐density sites from natal sites with low density. During the exponential population growth in the second period of the study, females dispersed mainly in a southern direction, towards low‐density areas. Our results demonstrate that natal dispersal patterns in the imperial eagle are sex‐dependent and are shaped by both intraspecific competition and conspecific attraction. The observed high philopatry implies the genetic uniqueness of the Pannonian population, making it of high conservation value.

## Graphical abstract

High philopatry, female‐biased natal dispersal distances, and density‐dependent dispersal patterns (with both conspecific attraction and competition avoidance) were found in the eastern imperial eagle. The study was conducted on the natal dispersal data obtained from 116 birds between 2011 and 2024 in the Pannonian region, using the combination of color‐ringing, GPS tracking, and DNA profiling methods.

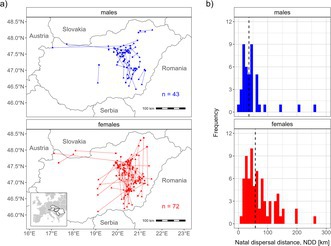

## 1 Introduction

Natal dispersal is defined as the movement of an individual from its place of birth to the site where it first attempts to breed (Greenwood 1980). Since it directly influences the distribution patterns of individuals, it has major implications for population dynamics, genetic structure, and colonisation potential, making its study important from both an ecological and a conservation perspective (Clark et al. 2004; Morrison and Wood 2009; Penteriani and Del Mar Delgado 2009; Morandini et al. 2019; Badia‐Boher et al. 2023).

Individual natal dispersal strategies are influenced by numerous external or “condition‐dependent” factors (such as conspecific density or habitat quality), as well as internal or “phenotype‐dependent” factors (including sex, age, and body condition) (Clobert et al. 2009; Delgado et al. 2010; Fattebert et al. 2019; McCaslin et al. 2020; Almasi et al. 2021; Passarotto et al. 2022). These factors may show varying impacts across dispersal stages and influence different dispersal metrics (propensity, distance, timing, and duration) in distinct ways (Clobert et al. 2004; Bowler and Benton 2005; Fattebert et al. 2019; Orgeret et al. 2023; Scherler et al. 2023). Consequently, natal dispersal strategy is a complex, plastic trait that is difficult to study (Bowler and Benton 2005; Clobert et al. 2009; Fattebert et al. 2019).

Sex bias in dispersal arises when the selective forces operating on dispersal traits are unbalanced between the sexes (Perrin and Mazalov 2000). Classical hypotheses suggest that the mating system is the main determinant of the direction of sex bias in dispersal (Greenwood 1980; Dobson 1982; Pusey 1987; Perrin and Mazalov 2000). However, it has been proposed that rather than the mating system itself, associated traits like sexual dimorphism in morphology, territoriality, and parental care drive the evolution of sex‐biased dispersal (Trochet et al. 2016), and social traits like kin cooperation can also influence dispersal patterns (Perrin and Goudet 2001). In raptors and birds in general, females are more likely to disperse from the natal population and typically exhibit longer dispersal distances than males (Greenwood 1980; Dobson 1982; Clarke et al. 1997; Trochet et al. 2016). This pattern has been linked to their typically monogamous mating system, where males acquire and defend the territory (Greenwood 1980). Since these tasks may be accomplished more successfully by being familiar with the area, males may benefit more from dispersing shorter distances (Greenwood 1980). As a response, females would have to disperse further to avoid inbreeding (Pusey 1987; Perrin and Mazalov 2000). While the classical theories are widely recognized as explanations for sex‐biased natal dispersal patterns, recent studies emphasize that the mechanisms underlying the sex bias in natal dispersal may be more complex and are still not well understood (Dobson 2013; Trochet et al. 2016; Li and Kokko 2019).

The relationship between dispersal and conspecific density is even less understood, with the driving factors appearing complex and studies providing inconsistent results on both the direction and the magnitude of density dependence, even within taxonomic groups (Jreidini and Green 2024). For instance, in raptors, both positive and negative density dependence, as well as no density dependence, have been observed (Katzner et al. 2012; McCaslin et al. 2020; Serrano et al. 2021). In the case of positive density dependence, individuals are more likely to disperse or disperse longer distances with increasing density, most probably to avoid increased intraspecific competition (Matthysen 2005; Jreidini and Green 2024). On the other hand, negative density dependence occurs when increasing density results in a lower frequency of dispersal or shorter dispersal distances. This may arise if the presence of conspecifics indicates an increased opportunity for mating or serves as a cue for high‐quality habitats (Matthysen 2005; Kim et al. 2009; Rodrigues and Johnstone 2014). The results on the density dependence of dispersal are shown to be significantly affected by the way density is measured and the dispersal metrics examined (Morton et al. 2018; Jreidini and Green 2024). Furthermore, most studies investigate the relationship between dispersal and density on a population level, as in migration between populations with different densities, and few examine within‐population dispersal in regard to local density patterns (Matthysen 2005; Jreidini and Green 2024). Studies also generally focus on how natal site density affects dispersal metrics, whereas the correlation between natal and breeding site densities is rarely examined (but see Penttinen et al. 2024).

Studying natal dispersal is particularly challenging in the case of raptors (Clark et al. 2004; Penteriani and Del Mar Delgado 2009). Firstly, they are typically elusive species with small population sizes, making them difficult to capture and mark in adequate numbers by conventional techniques of ringing, wing tags, or GPS tracking (Newton et al. 2016). An alternative marking method is genetic identification, when individuals are first DNA profiled as chicks, and later their genotypes are matched to the genotypes of breeding individuals to detect dispersal movements (Kylmänen et al. 2023; Penttinen et al. 2024). While the chicks need to be handled to obtain a DNA sample (usually blood or plucked feathers), birds in the breeding stage can be identified noninvasively by DNA profiling their shed feathers collected at their nest site, allowing for difficult‐to‐capture species to be sampled in great numbers (Horváth et al. 2005; Vili, Szabó, et al. 2013; Penttinen et al. 2024). Moreover, individuals can be sexed with high certainty from their DNA, which is an important aspect for raptors that usually display no plumage dimorphism, and size dimorphism is often not large enough to reliably differentiate the sexes (Newton 1979). Another issue is that raptors are highly mobile, meaning that long‐distance dispersal events can often remain undetected if the study area is limited or detection probability decreases with distance from the core study area, which is usually the case when birds are marked with rings or wing tags (Chadœuf et al. 2018). GPS tracking overcomes this issue, but it is a more costly method, usually allowing for much smaller sample sizes (Serrano 2018). The difficulty of studying raptors is further compounded by the fact that many species have delayed maturity, with a floater period of several years (Newton 1979). This necessitates long‐term studies to investigate natal dispersal, which require a significant amount of time, finances, and human effort. Delayed maturity also means that equipment failure of GPS trackers or the loss of color rings and wing tags may happen sooner than the birds settle for breeding, further decreasing the number of individuals for which natal dispersal distances (NDDs) can be recorded (Serrano 2018). Consequently, using multiple marking methods may be beneficial when studying natal dispersal in raptors.

Here we studied natal dispersal in the eastern imperial eagle *Aquila heliaca* (hereafter: imperial eagle), in the Pannonian breeding population. This large‐sized, long‐lived raptor species prefers the forest‐steppe habitats of Eurasia, where patches of trees provide nesting places next to open grasslands suitable for foraging (del Hoyo et al. 1994; Horváth et al. 2010). Its distribution spans from Austria in the west to the Trans Baikal region of Russia in the east and from the Southern‐Ural Mountains of Russia in the north to Turkey in the south (Horváth et al. 2002; Demerdzhiev et al. 2011; Karyakin 2020). The small, isolated, western populations in Central Europe, the Balkans, and Turkey are mostly sedentary, while the large eastern populations are migrants, wintering in the Middle East, South‐East Asia, and North‐East Africa (Horváth et al. 2002; Demerdzhiev et al. 2011). The global population of the imperial eagle is estimated at less than 10,000 mature individuals, making it a globally vulnerable species (BirdLife International 2019). About two‐thirds of its global population is found in Russia and Kazakhstan (Karyakin 2020). A significant number of breeding pairs form a compact, geographically isolated population in the Pannonian Region, in the westernmost part of the species' distribution (Demerdzhiev et al. 2011; Karyakin 2020).

There is currently only scarce published data on NDDs in the imperial eagle (Korepov 2023; Rymešová et al. 2023), with no substantial data on sex‐specific, nor any data on density‐dependent patterns. Improved knowledge of these topics would greatly benefit the conservation of this species by offering insights into its colonization potential (González et al. 2006) and the extent of genetic exchange among its geographically scattered populations. Exploring the relationship between dispersal and density could facilitate our understanding of previously observed colonization patterns. Furthermore, identifying any sex‐specific patterns could shed light on potential spatial variation in sex ratios, an important aspect considering previous evidence on sex‐dependent survival rates (Zsinka, Pásztory‐Kovács, et al. 2024). Additionally, knowledge of natal dispersal and associated migration rates would enhance the accuracy of models predicting future population trajectories for this vulnerable species (Badia‐Boher et al. 2024).

We investigated natal dispersal patterns using recapture data obtained through color‐ringing, GPS tracking, and DNA profiling. We studied the NDD (i.e., the distance between the natal nest and the first documented breeding location) of birds in relation to their sex and density at the natal and breeding sites. We hypothesised that females would disperse further, as this aligns with the general pattern observed in birds. Furthermore, we examined the relationship between NDD and natal density and investigated whether the eagles dispersed to higher or lower density areas compared to their natal site. We also examined the directional patterns of natal dispersal movements.

## 2 Materials and Methods

### 2.1 Study Population

We studied the natal dispersal of imperial eagles in the Pannonian Region between 2011 and 2024. The size of this nonmigratory population was estimated at 356–381 nesting pairs as of 2019 (Karyakin 2020), with 276 pairs nesting in East Hungary and 11 in West Hungary (Horváth et al. 2020), 45–50 in East Slovakia (Danko and Mihók 2020), 21 in West Slovakia (Chavko et al. 2022), 20–23 in East Austria (Schmidt and Horal 2018) and 6–8 in the Southern Czech Republic (Schmidt and Horal 2018), 3 pairs in North Serbia and one in West Romania (Horváth 2022). The eastern (East Hungary, East Slovakia, West Romania and North Serbia) and western (West Hungary, West Slovakia, East Austria and the Southern Czech Republic) breeding nuclei of the population are separated by 100 km, with mild genetic differentiation between these two subpopulations (Vili et al. 2009).

In the 1980s, the imperial eagle occupied almost exclusively mountainous forest habitats (200–700 m a.s.l.) in the Pannonian Region, where it could find refuge from the intensive persecution occurring in the agricultural lowlands (Bagyura et al. 2002; Horváth et al. 2010). Following the decrease in persecution levels, the number of imperial eagles has increased exponentially, and their distribution has expanded first to the foothill plains, then to the nearby lowlands (Horváth et al. 2011). In Hungary, this population expansion occurred mainly in a southern direction, from the North Hungarian Mountains towards the Hungarian Great Plain (Horváth et al. 2011). Now, the vast majority of pairs in the Pannonian population breed in lowland agricultural areas (80–120 m a.s.l.) (Schmidt and Horal 2018; Chavko et al. 2022; Horváth 2022). In the lowlands, birds usually forage within a 3–8 km radius of the nest on agricultural fields and grasslands while in the mountainous habitats, foraging sites can be as far as 10–15 km from the nesting site (Horváth et al. 2010). Between 2011 and 2014, the population in Hungary went through a stagnating phase, most probably due to the high rates of poisoning observed during this time (Horváth 2022; Zsinka, Pásztory‐Kovács, et al. 2024). From 2015, after poisoning levels have decreased, the population has again started growing exponentially and has tripled its size by 2022 (Deák et al. 2021; Horváth 2022; Zsinka, Pásztory‐Kovács, et al. 2024).

Most imperial eagles start breeding in their third or fourth calendar year (abbreviated as “cy”, where the first calendar year is the year of hatching) (Horváth 2022) until which they act as floaters, often exploring areas hundreds or thousands of kilometres from their natal place (Horváth 2022; Rymešová et al. 2023). Despite their high mobility, most floater movements are restricted to their natal population, suggesting high natal philopatry (Prommer et al. 2015; Rymešová et al. 2023). Previous studies based on genetic identification also indicate high breeding philopatry (Rudnick et al. 2005; Vili, Szabó, et al. 2013; Zsinka, Pásztory‐Kovács, et al. 2024). Adult birds display only minor sexual dimorphism in morphology: females are slightly larger than males. However, they have markedly different sex roles during the breeding, as females do most of the incubation while males forage for food (Dobrev 2009; Horváth 2022).

Imperial eagle nesting sites in Hungary have been monitored annually by the Hungarian Imperial Eagle Working Group, which has been operated by MME BirdLife Hungary and the Hungarian national park directorates since 1980. Each year, the locations of nests are recorded (WGS84 coordinate system, five decimal accuracy), along with the breeding success and additional observations on the age and identity of the breeding pair. The monitoring programs were well‐organized and had wide coverage during the study period; therefore, the number of known nests is estimated to account for 95% of the total number of nests in the country (Horváth et al. 2011, 2020).

### 2.2 Monitoring of Natal Dispersal

We monitored the natal dispersal of imperial eagle chicks hatched and marked in Hungary between 2011 and 2022 in the frame of the Helicon LIFE (2012–2016, LIFE10NAT/HU/019) and PannonEagle LIFE (2017–2023, LIFE15 NAT/HU/000902) projects. Three types of marking were used for individual identification: ringing (metal ornithological and plastic color‐rings), GPS tracking devices, and DNA profiling. Chicks were either (i) only ringed, (ii) ringed and fitted with a GPS tracker, (iii) ringed and DNA profiled, or (iv) all three types of marking were applied. For molecular sexing, DNA samples were also collected from ringed and GPS‐tracked chicks, which were not DNA profiled.

We defined an individual's NDD as the great circle distance in km between its natal nest and the nest where it had its first recorded breeding attempt with successful egg‐laying (González et al. 2006). The first recorded breeding site is most likely the same as or close to the actual first breeding site since breeding dispersal is rare and usually short distance (Rudnick et al. 2005; Vili, Szabó, et al. 2013; Zsinka, Pásztory‐Kovács, et al. 2024).

### 2.3 Color‐Ringing

Altogether 1660 imperial eagle chicks were ringed between 2011 and 2022 at the age of 4–9 weeks and were tagged with both a metal ornithological ring and a colored plastic ring (black code on white background).

All ringing and encounter data were obtained between June 2011 and April 2024 from the Hungarian Bird Ringing Databank operated by the Hungarian Bird Ringing Centre of MME BirdLife Hungary. The coordinates of hatching and encounter locations were recorded in the WGS84 projection system with a five‐decimal‐place accuracy.

To differentiate actual natal dispersal movement (when the encounter location designates a breeding site) from floater movement (explorative movement of immature birds between areas with no breeding attempt), we only considered movements where (i) the encountered bird was at least the age of 3 cy (earliest recorded age of breeding in imperial eagles, Schmidt and Horal 2018), (ii) the encounter location could be assigned to a known nesting site, (iii) observations on the age or identity of breeding birds at the nest site confirmed the breeding status, and (iv) egg‐laying occurred at the designated breeding site in the year of encounter.

### 2.4 GPS Tracking

In addition to ringing, between 2011 and 2022, 71 chicks at the age of 7–10 weeks were also equipped with solar‐powered GPS backpack transmitters (Microwave Telemetry, Ecotone Telemetry or Ornitela Telemetry). The mean body weight of the tagged chicks was 2.9 kg, and the weight of the transmitter applied (29–70 g) was less than 2% of the birds' body weight (Kenward 2000). Data registered by the transmitters were stored in the Movebank.org online database, within different studies of MME BirdLife Hungary.

We used GPS tracking data recorded until 30 September 2024. To decide whether a GPS‐tracked bird has entered the breeding stage, we examined the movement patterns of birds during each breeding season starting from their third calendar year. Incubating females exhibit a specific movement pattern, where most of their movements are restricted to the nest site, with occasional movements to other parts of the territory. Males also display restricted movement concentrated around the nest site when breeding. Besides, the breeding attempts of GPS‐tracked birds were monitored in the field by the Hungarian Imperial Eagle Working Group. Similarly to ringed birds, we only recorded the movement of tracked birds as natal dispersal if at least egg‐laying occurred at the breeding site.

### 2.5 DNA Profiling

We used genetic monitoring as a third method of studying natal dispersal: we searched for breeding birds and chicks with matching microsatellite genotypes. We analysed the DNA samples (armpit feathers) of 631 chicks ringed between 2011 and 2018, along with the DNA samples of breeding birds (shed feathers found under the nests of breeding pairs) collected from 336 territories between 2013 and 2022. Chicks and breeding adults were sampled in different timeframes, as we expected the chicks hatched in 2011 to start breeding in 2013 (their third calendar year) at the earliest. While we could not verify in all cases through parentage analysis that the shed feathers collected in a given year belonged to the breeding pair, feathers at the nest site almost exclusively originate from the resident pair (Pásztory‐Kovács's personal communication), and only feathers shed in the same year when collected are in a suitable condition for DNA analysis (Vili, Nemesházi, et al. 2013).

Laboratory procedures of sample preparation, DNA extraction, molecular sexing, and individual identification using the 9‐loci microsatellite set are described in detail in Zsinka, Pásztory‐Kovács, et al. (2024). We used a two‐step method to find matches between breeding adults and chicks and applied strict rules of identity to reduce the probability of false matches. In the first screening, we sexed both the chicks and the breeding birds (primers CHD‐i16F/CHD‐i16R, Suh et al. 2011) and individually identified them using nine microsatellite loci: Aa02, Aa35, Aa36, Aa39, Aa43 (Martínez‐Cruz et al. 2002); Hal04, Hal10 (Hailer et al. 2005); IEAAAG09, IEAAAG11 (Busch et al. 2005). During this first screening, we only accepted a chick and a breeding bird as the same individual if they had matching genotypes on all nine loci and matching sex. To increase the sample size without compromising the reliability of matches, we did a second screening for those pairs of chicks and breeding adults that were genotyped successfully only on eight or seven loci but showed a full match on these loci along with matching sex. These individuals were genotyped on an additional eight loci: Aa26, Aa41, Aa43, Aa53, Aa57 (Martínez‐Cruz et al. 2002); AQJ10, AQJ22 (Sato et al. 2015); AQJ120 (Naito‐Liederbach et al. 2021). For these loci, we used the same PCR mix described in Zsinka, Pásztory‐Kovács, et al. 2024 and a modified version of the PCR program published in Martínez‐Cruz et al. 2002: 95°C for 2 min; 17 cycles with a touchdown scheme: denaturation at 95°C for 30 s, annealing at 66°C–50°C for 30 s (−1°C/cycle), elongation at 72°C for 30 s; 21 cycles of denaturation at 95°C for 30 s, annealing at 50°C for 30 s, and elongation at 72°C for 30 s; final elongation at 72°C for 7 min (Zsinka, Vili, et al. 2024). Similarly to the first screening, we only accepted pairs of chicks and breeding adults as identical if they showed a full match on all the genotyped loci.

To assess the reliability of the marker sets for individual identification, we calculated the probability of identity values corrected for the presence of siblings in the population (PI_SIB_, Waits et al. 2001) for both the 9‐ and 17‐loci sets using GenAlEx v.6.503 (Peakall and Smouse 2006).

### 2.6 Variables of Natal Dispersal

We carried out spatial computations in R v.4.2.1. (R Core Team 2023) with the “sf” package (v. 1.0‐12, Pebesma 2018).

We aimed to define density as a continuous variable which describes the local density of active nests. As a measure, we used the reciprocal area (1/km^2^) of the truncated Thiessen (Voronoi) polygon (Mcleod et al. 2002; Schlicht et al. 2014) created around each active nest. Polygons were truncated at a maximum radius of 12.7 km, the average nearest neighbor distance value calculated for the Hungarian population at a much lower density, with no observable saturation or density‐dependent effect (1989–2006, Horváth et al. 2014). We only calculated local density for 2011–2022, the period of highly intensive nest monitoring in the frame of the HELICON and PannonEagle LIFE projects. While we only calculated local density for nests in Hungary, we also accounted for the presence of nests in the neighboring countries (where monitoring was similarly intensive as in Hungary). When a nest had a Thiessen polygon intersecting the country border, either we corrected its polygon for nests in the neighboring country (Austrian, Romanian, and Serbian border: nest coordinates provided by M. Schmidt, Z. Hegyeli, and M. Ružić, respectively), or we excluded the nest from density calculations (Slovakian border).

For each individual, we defined breeding density as the local density at their first documented breeding nest in their expected year of recruitment. The definition of the expected year of recruitment was based on the median age when imperial eagles started breeding (4cy, calculated from the GPS‐tracked birds in this study). For birds that were first observed breeding at the age of 4 cy or younger, the expected year of recruitment was the year when they were first observed breeding. For birds that were first observed breeding older than 4 cy, the expected year of recruitment was the first year when the bird was at least 4 cy old, the territory had already existed, and it was not known to be occupied by another bird of the same sex.

We defined natal density for each individual as the local density at their natal nest in their expected year of recruitment (current density at the natal site, Kim et al. 2009; Penttinen et al. 2024). We used the same year for both natal and breeding density to account for the changes in population density over the years.

### 2.7 Statistical Analysis

We used R v.4.2.1. (R Core Team 2023) for all statistical analyses. We calculated the 95% confidence intervals for the median NDDs of males and females by inverting the sign test (Hollander et al. 2013) using package “BSDA” (v.1.2.2, Arnholt and Evans 2023).

We used package “lmerTest” (v. 3.1‐3, Kuznetsova et al. 2017) to fit general linear mixed models.

We tested the change in median local density over the years in a model with *density* (log‐transformed) as the response variable, *year* as the explanatory variable (continuous) and *territory ID* as a random intercept.

To investigate the relationships between the natal dispersal variables, we constructed two sets of general linear mixed models.

In the first set of models, *NDD* was the response variable (log‐transformed), while the explanatory variables were *sex* (male as the reference category), *natal density* (log‐transformed) and its quadratic term (describing a U‐shaped pattern, which may occur with simultaneous positive and negative density‐dependence, Kim et al. 2009) and *natal period* (two‐level factor, where natal years 2012–2014 mark a period of population stagnation and natal years 2015–2018 correspond to population increase). *Natal territory ID* and *n* *atal year* were included in the models as crossed random intercepts to account for the correlation between individuals originating from the same territory and hatching in the same year.

We constructed another set of models with *density difference* (*log(breeding density*))—(*log*(*natal density*)) as the response variable, *sex* (male as the reference category), *NDD* (log‐transformed) and its quadratic term, *natal density* (log‐transformed) and its quadratic term, and *natal period* (two‐level factor, 2012–2014 as reference) as explanatory variables, and *natal territory ID* and *natal year* as crossed random intercepts. Additionally, we tested the Pearson correlation between *breeding density* and *natal density* for males and females (*p*‐values adjusted with the Bonferroni–Holm method). We also calculated the mode of the breeding density distribution based on kernel density estimates.

Exploratory analysis and model diagnostics revealed that the best fit to the linear models and homogeneity of variance were achieved by log transforming both the response and the explanatory variables (*NDD* and *natal density*). This also allowed for keeping variables consistent among models of *NDD* and *density difference*. We also scaled all continuous explanatory variables. In all cases, we initially built models with two‐way interactions between the main effects and excluded insignificant interactions and quadratic terms (*p* ≥ 0.05) from the reduced models but retained all main effects irrespective of their significance. We investigated model fit based on visual inspection of residuals, outliers and the normality of the random effects.

We also calculated the direction of natal dispersal movements using the “lwgeom” package (v.0.2‐14, Pebesma 2024). To explore any heterogeneity in the direction of natal dispersal movements, we used Rayleigh tests (package “circular” v.0.5‐1, Agostinelli and Lund 2024) for both male and female movements and movements in the two periods (natal years 2012–2014 and 2015–2020).

We made the figures using “ggplot2” (v.3.5.1, Wickham 2016), “sjPlot” (v.2.8.17, Lüdecke 2024), “ggspatial” (v.1.1.9, Dunnington 2023), “RColorBrewer” (v.1.1‐3, Neuwirth 2022) and “gridExtra” (v.2.3, Auguie 2017). We obtained the country polygons via package “rnaturalearth” (v.1.0.1, Massicotte and South 2023).

## 3 Results

### 3.1 Color‐Ringing Data

336 of the 1660 ringed chicks were encountered and identified based on their ring ID at least once until April 2024 (Figure S1). Altogether, 468 encounters were recorded, of which 137 were recoveries of dead or injured birds, 2 were live recaptures, and 329 were resightings. In the case of 261 resightings, the birds were identified based on their color‐ring ID, while identification based on the metal ring ID occurred only in 67 cases (presumably when the color‐ring became damaged or lost). Most observations came from Hungary (348) and the neighboring countries of the Pannonian region (92). Only 28 encounters were recorded outside of the Pannonian region (one bird observed in Turkey, one in Italy, one in Poland, one in Macedonia, one in the Netherlands and one both in Finland and the Netherlands). Of these individuals encountered outside the Pannonian population, only two were at least 3cy, and neither of them was observed in a breeding population. In summary, no imperial eagle ringed in Hungary was observed breeding in another population.

### 3.2 GPS Tracking Data

Of the 71 GPS‐tracked birds, 33 were male, 31 were female, and 7 were of unknown sex (Figure S1). Only 34 birds reached the 3 cy age of maturation, and the oldest age until an individual was followed was 10cy (after which equipment failure occurred).

### 3.3 DNA Profiling Data

We DNA profiled 631 chicks (298 males, 300 females, and 33 of unknown sex) hatched between 2011 and 2018 (Figure S1). Additionally, we genotyped 1786 shed feathers between 2013 and 2022, which belonged to 510 breeding birds (162 males, 334 females, and 14 of unknown sex) (Figure S1). The probability of identity corrected for the presence of siblings in the population (PI_SIB_) confirmed that the markers used were reliable for individual identification, even in the case of the smaller, 9‐loci marker set (PI_SIB_ = 3.5 × 10^−6^ for the 17‐loci set and PI_SIB_ = 5.8 × 10^−4^ for the 9‐loci set).

### 3.4 Recorded Natal Dispersal Events

Using data from 2011 to 2024, we managed to determine the NDD of 116 imperial eagles hatched between 2012 and 2020: 43 males, 72 females, and one with unknown sex. Most of these natal dispersal events were detected via only one of the three identification methods (68 from DNA profiling, 23 from ringing and 13 from GPS tracking), but some events were detected by multiple methods (six with both DNA profiling and ringing, five with both DNA profiling and GPS tracking and one with both ringing and GPS tracking). We found no contradiction between the matches reported by the three methods.

For GPS‐tracked birds, the age at first breeding was precisely known, with a median of 4 cy for all birds (range 3 cy–6 cy, *n* = 19), 4 cy for males (range 4 cy–6 cy, *n* = 7) and 4.5 cy for females (range 3 cy–6 cy, *n* = 12). In contrast, the first detected breeding for ringed and DNA‐profiled birds may not have been their actual first breeding. Consequently, age at the first detected breeding was higher for birds surveyed using ringing or DNA profiling data (ringing: median 6 cy, range 3 cy–11 cy, *n* = 30; DNA: median 5 cy, range 3 cy–9 cy, *n* = 79).

**FIGURE 1:**
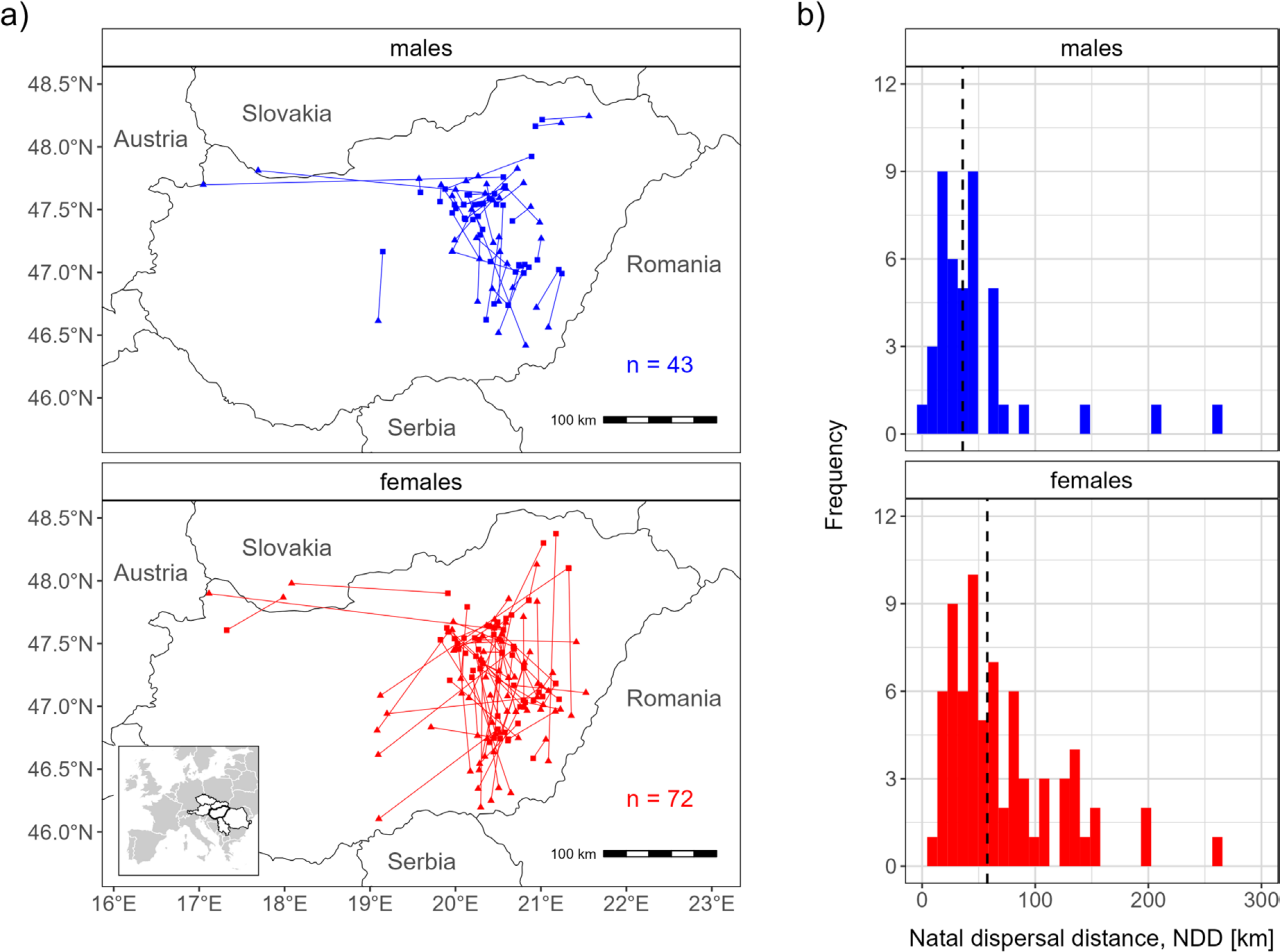
Natal dispersal movements (a) and distribution of natal dispersal distances (b) of eastern imperial eagles hatched in Hungary between 2011 and 2022. On (a), squares denote the natal site and triangles the first detected breeding site; on (b) vertical dashed lines indicate the median distances. Inset highlights the countries occupied by the Pannonian population.

GPS tracking data revealed three birds (two females and one male, 2.6%), which started breeding in one of the neighboring countries, in the western part of Slovakia. Concerning the dispersal between the eastern and western parts of the Pannonian population, five birds that hatched in East Hungary (4.3%) dispersed to the western part (one male, one female and one bird of unknown sex to West Hungary; one male and one female to West Slovakia). No dispersal was detected from West Hungary to the eastern part of the population (Figure 1).

### 3.5 Natal Dispersal Distance in Relation to Sex, Natal Density and Natal Year

The median NDD was 35.9 km for males (95% CI 22.9—45.6 km, range 1.8–262.8 km, *n* = 43) and 57.6 km for females (95% CI 46.4—69.7 km, range 12.3–259.6 km, *n* = 72) (Figure 1). Only those birds for which natal density could be determined (39 males and 62 females) were included in the linear mixed models with *log* (*NDD*) as the response variable. NDDs were significantly longer for females than for males (Table 1). We found no significant linear or quadratic effect of *log* (*natal density*) or *natal period*, and none of the two‐way interactions were significant.

**TABLE 1:** Estimates of the general linear mixed model investigating the relationship of natal dispersal distance (NDD, log‐transformed) with sex (“male” as reference), natal density (log‐transformed, scaled) and natal period (two‐level factor, “2012–2014” as reference) in 39 male and 62 female eastern imperial eagles hatched in Hungary between 2012 and 2018. Natal territory ID and natal year (categorical) were set as crossed random intercepts. Effects in bold were significant (*p* < 0.05).

Response variable: log (NDD)					
Explanatory variables	Estimate	SE	df	*t*	*p*
**Intercept**	**3.333**	**0.165**	**4.868**	**20.18**	**< 0.0001**
**Sex (female)**	**0.546**	**0.157**	**70.74**	**3.479**	**0.0009**
log (natal density)	−0.045	0.080	60.09	−0.557	0.5797
Natal period (2015–2018)	0.274	0.199	3.251	1.379	0.2551

Random effects	SD				
Natal territory ID	0.412				
Natal year	0.161				
Residual	0.633				

There was one outlier in the models, a male who settled in the adjacent territory of its natal site (1.8 km) for breeding. We repeated the analysis excluding this record and could draw the same conclusions (Table S1).

### 3.6 Density Difference in Relation to Sex, Natal Density and NDD

**FIGURE 2:**
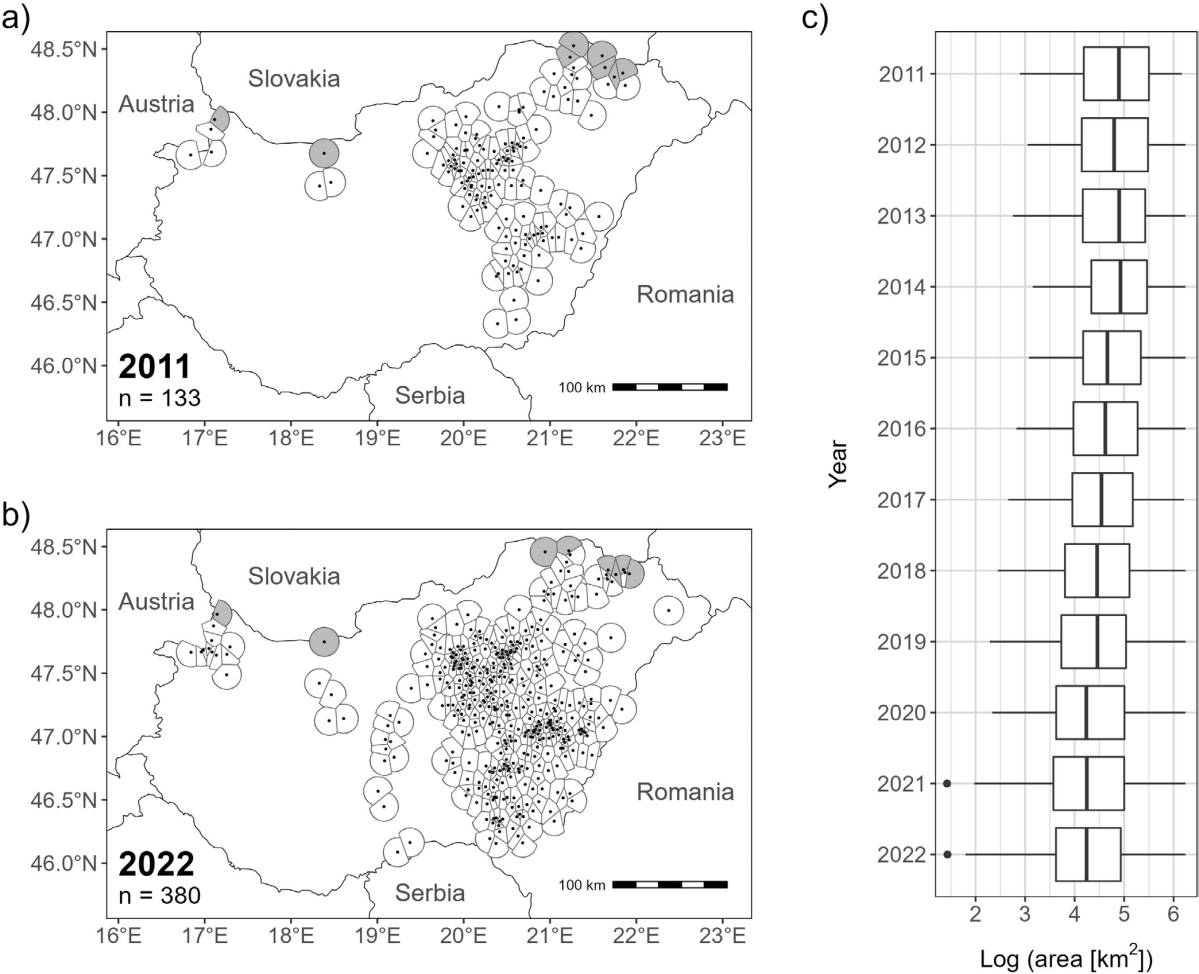
Thiessen polygons of eastern imperial eagle nests sampled between 2011 and 2022 in Hungary. Maps show the first (a) and final (b) sampling year, nests are marked with black dots. Thiessen polygons were truncated at a maximum radius of 12.7 km. Nests with grey polygons were excluded from the analyses including local density since exact locations of nests in Slovakia were not known. Log transformed area of the Thiessen polygons in each sampled year are shown on (c). The reciprocal of an individual's Thiessen polygon area was used as a measure of local density.

Median local density calculated for all the active nests increased over the study period from 0.007 km^−2^ in 2011 to 0.014 km^−2^ in 2022 (*β* = 0.089, SE = 0.002, *p* < 0.001, Figure 2). The minimum number of recorded territories in a year was 133 in 2011, and the maximum was 380 in 2022.

Only those birds for which we could determine both the breeding and the natal density (39 males and 62 females) were included in the Pearson correlation test between natal and breeding density. In the case of males, natal site and breeding site density were significantly positively correlated (*r* = 0.455, *p* = 0.0073), while for females, we found no significant correlation between the two densities (*r* = 0.161, *p* = 0.2110).

**FIGURE 3:**
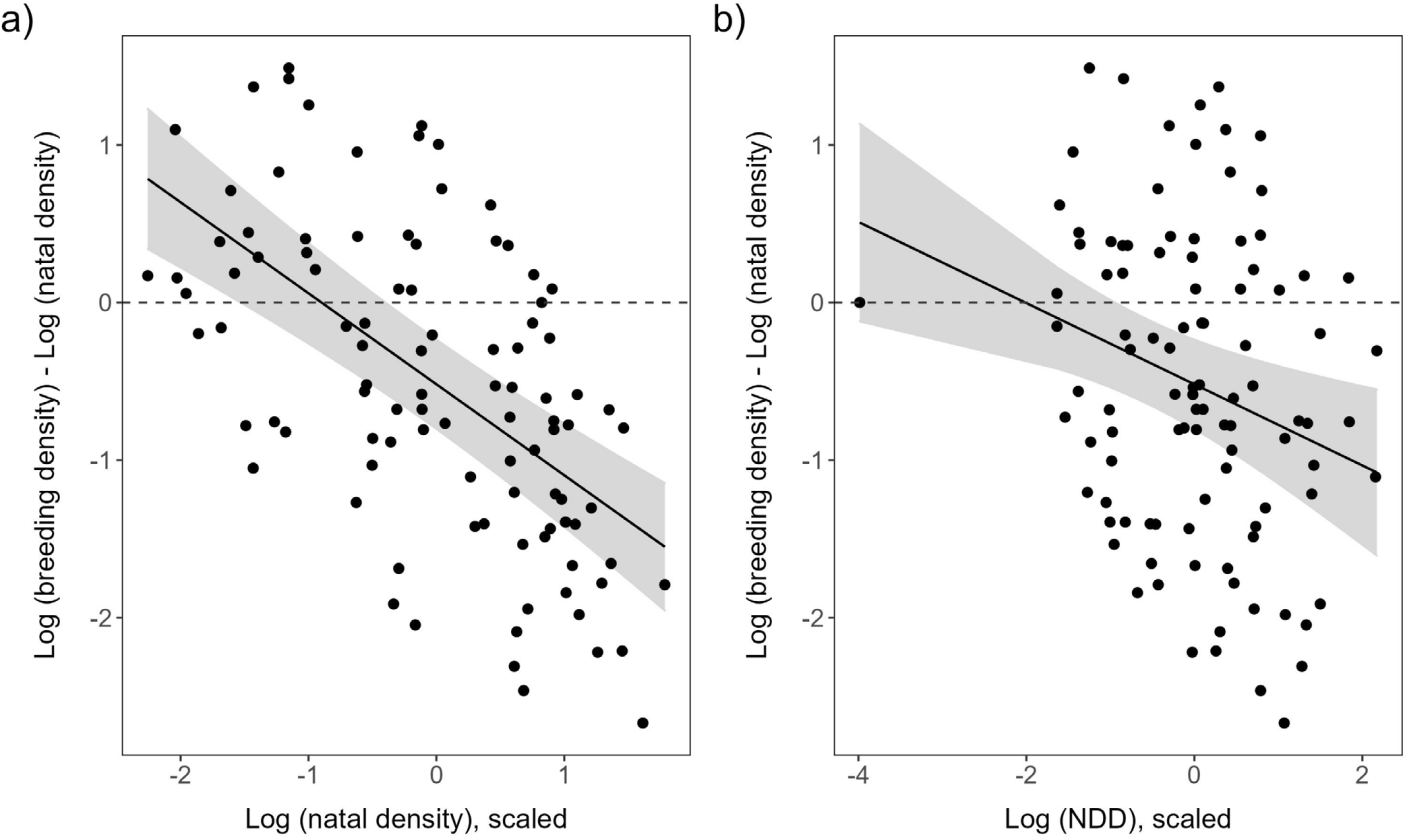
Difference between natal site and breeding site density in relation to natal site density (a) and natal dispersal distance (b) in eastern imperial eagles hatched in Hungary between 2012 and 2018. Slopes and 95% confidence intervals were estimated from the linear mixed model in Table 2. The horizontal dashed line on both (a) and (b) indicate zero density difference (in such a dispersal event, natal and breeding nest had the same density value).

We could use the data of the same 39 males and 62 females in the linear mixed models with *density difference* (*log* (*breeding density*)—*log* (*natal density*)) as the response variable. As shown by the intercept of the model, the mean *density difference* was significantly lower than zero (Table 2), indicating that, on average, both males and females dispersed to lower‐density territories compared to their natal sites (Figure 3a,b). Both *log* (*natal density*) and *log* (*NDD*) showed a significant negative relationship with *density difference* (Table 2, Figure 3a,b). We found no significant effect of *sex* or *natal period*, and neither the quadratic effect of *log* (*natal density*) nor *log* (*NDD*), nor any of the two‐way interactions were significant. We repeated the analysis excluding the unusually short dispersal distance of a male, and we obtained similar results (Table S2).

**TABLE 2:** Estimates of the general linear mixed model investigating the relationship of density difference (log (breeding density)—log (natal density)) with sex (“male” as reference), natal density (log‐transformed, scaled), natal dispersal distance (NDD, log‐transformed, scaled) and natal period (two‐level factor, “2012–2014” as reference) in 39 male and 62 female eastern imperial eagles hatched in Hungary between 2012 and 2018. Natal territory ID and natal year (categorical) were set as crossed random intercepts. Effects in bold were significant (*p* < 0.05).

Response variable: density difference (log (breeding density)—log (natal density))					
Explanatory variables	Estimate	SE	df	*t*	*p*
**Intercept**	**−0.519**	**0.143**	**90.49**	**−3.637**	**0.0005**
Sex (female)	0.062	0.169	93.62	0.364	0.7166
**Log (natal density)**	**−0.578**	**0.079**	**72.65**	**−7.345**	**< 0.0001**
**Log (NDD)**	**−0.258**	**0.084**	**95.74**	**−3.084**	**0.0027**
Natal period (2015–2018)	−0.112	0.159	95.99	−0.704	0.4831

Random effects	SD				
Natal territory ID	0.236				
Natal year	0.000				
Residual	0.729				

The model predicted zero *density difference* at the density value of 0.009 km^−2^ that was very close to the mode of the breeding densities (0.007 km^−2^, Figure S2).

### 3.7 Direction of Natal Dispersal Movements

**FIGURE 4:**
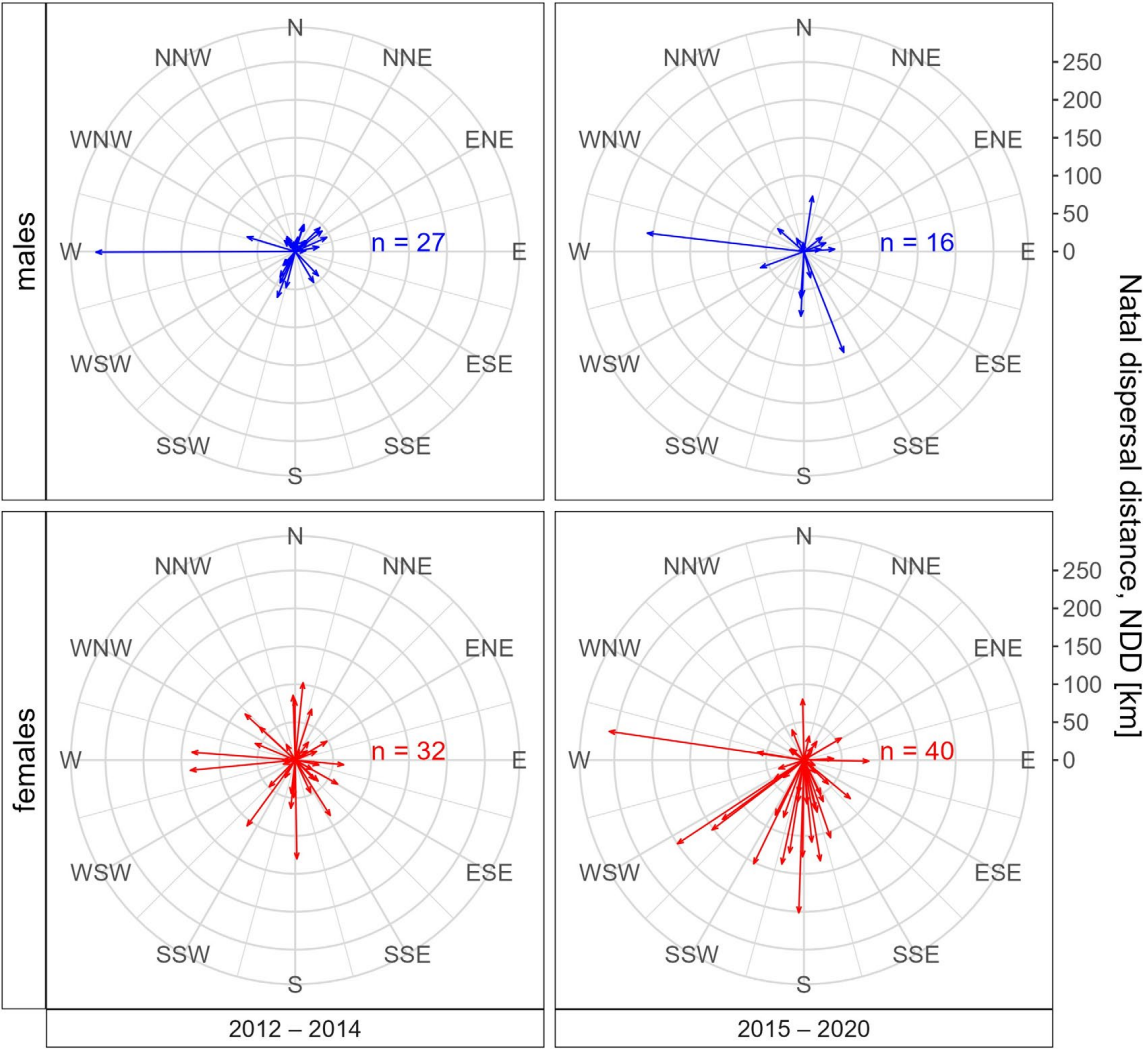
Natal dispersal directions and distances of eastern imperial eagles hatched in Hungary in 2012–2014 and 2015–2020. Arrow length indicates distance; abbreviations indicate direction.

We found no deviation from uniformity concerning the direction of natal dispersal movements among birds hatched in the first period of the study, 2012–2014 (Rayleigh tests, males: *r* = 0.218, *p* = 0.279, *n* = 27; females: *r* = 0.055, *p* = 0.908, *n* = 32). However, the natal dispersal directions of females hatched in the second period (2015–2020) showed a significant deviation from uniformity, with most females dispersing towards the south at a mean angle of 183.5° (Rayleigh tests, males: *r* = 0.130, *p* = 0.768, *n* = 16; females: *r* = 0.370, *p* = 0.004, *n* = 40) (Figure 4). Even though NDDs seem longer in the second period, the model with *log* (*NDD*) as response variable showed no significant effect of *natal period* (see Table 1).

## 4 Discussion

Using the combination of color ringing, GPS tracking, and DNA profiling, we revealed that natal dispersal in the imperial eagle is female‐biased, and we also found implications for both positive and negative density dependence: birds generally settled in lower‐density sites compared to their natal site but moved to higher‐density sites when their natal‐site density was low.

### 4.1 Female‐Biased Natal Dispersal

We found that imperial eagle females dispersed further than males, as expected from theory and empirical evidence from other birds of prey (Greenwood 1980; Trochet et al. 2016; Nemesházi et al. 2018; Li and Kokko 2019; Murphy et al. 2019; Penttinen et al. 2024; Whitfield et al. 2024). The few NDD values previously reported for the imperial eagle (Korepov 2023; Rymešová et al. 2023) are similar to the values we obtained, but these studies could not make a meaningful comparison between the sexes due to their limited sample sizes.

Sex‐biased natal dispersal can be an important mechanism of inbreeding avoidance (Li and Kokko 2019), which is especially crucial in species like the imperial eagle, where small population size and long‐term genetic monogamy coupled with high longevity and strong philopatry result in a high number of siblings within a population (Rudnick et al. 2005; Vili, Szabó, et al. 2013). We argue that the longer dispersal distances of females helped maintain the Pannonian population's relatively high genetic variability despite the previous bottleneck (Vili et al. 2009). On the other hand, the shorter distances of males limit the colonization potential and speed in this population.

Sex‐biased natal dispersal has the potential to introduce spatial variation in sex ratios and, therefore, elevate the extinction risk of small, fragmented populations (Dale 2001). Our results suggest that more birds might disperse from the larger eastern to the smaller western subpopulation than *vice versa* (although we marked a significantly larger proportion of chicks in the East than in the West). While we found no evidence of female bias among the individuals dispersing from East to West, the longer NDD of females implies the possibility that most of these long‐distance dispersers are females. This could lead to a female‐biased sex ratio in the West, which may be further enhanced by male‐biased adult mortality due to poisoning (Zsinka, Pásztory‐Kovács, et al. 2024). Considering monogamy and the important role of males in providing food for their young, such spatial inhomogeneity in sex ratios is expected to decrease reproductive output at the population level.

### 4.2 Density‐Dependent Natal Dispersal

Our results imply that natal dispersal patterns in the imperial eagle are shaped by both competition avoidance and conspecific attraction (Penttinen et al. 2024). In territorial birds, such as the imperial eagle, high conspecific density may not only decrease survival and breeding success through resource depletion, but also via elevated frequencies of territorial intrusions and fights (Heuck et al. 2017). These effects can lead to a positive relationship between density and dispersive tendency (Matthysen 2005; Jreidini and Green 2024). In line with this, we found that most birds hatched at medium‐ and high‐density areas chose lower density breeding sites compared to their natal site.

On the other hand, we also found indications of negative density dependence, as birds that hatched in low‐density areas settled mainly in higher‐density areas, showing evidence for conspecific attraction. Conspecific presence can serve as a cue for suitable or high‐quality habitats and signal an increased chance for mate acquisition (Matthysen 2005; Kim et al. 2009; Rodrigues and Johnstone 2014; Ferrer et al. 2015). By relying on social cues, imperial eagle floaters may reduce the chance of choosing an unsuitable territory, which would result in a high cost considering their strong breeding philopatry.

Based on our model predictions, birds originating from a local density of 0.009 km^−2^ (~111 km^2^ polygon area) tend to disperse equally towards higher and lower densities; additionally, most birds settle in territories with approximately this density (Figure S2). Thus, we argue that it may represent an optimal trade‐off: minimizing the risk of settling in a territory with limited resources or mating opportunities while incurring relatively low competition.

Since movements are directed from territories with extreme densities towards those with low‐medium density, dispersal is expected to homogenise breeding pair density. On the other hand, most individuals originate from high‐density—thus, presumably good‐quality—territories and males especially try to settle close to their natal sites, resulting in density heterogeneity. This highlights the two‐way relationship between density and dispersal: not only does density influence dispersal, but dispersal also shapes density (Jreidini and Green 2024). Historical reasons may also help explain the highest densities in the core breeding areas at the foothills, as these were occupied first by this exponentially growing population (Horváth et al. 2011).

Even in the high‐density, presumably high‐quality territories, carrying capacity has not yet been reached since settlement via territory partitioning still occurs. This ongoing partitioning of high‐quality territories indicates that territory sizes in the imperial eagle appear to align more closely with an ideal free distribution (Zammarelli et al. 2024) rather than an ideal despotic one (Zimmerman et al. 2003). However, evaluating the relationship between density and fitness remains a task for future research.

The significant positive correlation between breeding site and natal site density in males but not in females complements our results on the sex‐biased NDDs in this species; since males disperse shorter distances than females, their breeding and natal site densities are expected to be more similar. Our results also indicated that the longer NDDs belong to individuals moving from high‐density breeding territories to low‐density peripheral areas. It would be interesting to study whether these longer dispersal distances are associated with lower fitness (Forero et al. 2002; Bloom et al. 2011; Dykstra et al. 2019).

How density is defined can influence the conclusions drawn on its effect (Morton et al. 2018). In this study, we aimed to measure local density on a continuous scale and defined it as the reciprocal area of Thiessen polygons drawn around the nest. It is important to note that the area covered by the Thiessen polygon of a breeding bird's nest is generally a good indicator, but does not necessarily correspond completely to the area of its territory (Mcleod et al. 2002; Schlicht et al. 2014). While it was most probably the case in high‐density areas, in low‐density areas, actual territory sizes may have been smaller than indicated by the Thiessen polygons.

### 4.3 Temporal Changes in Dispersal Patterns

We found that birds hatched in the first period of the study showed no preferred direction of natal dispersal, whereas females hatched in the second period mainly dispersed in a southern direction. We only detected this directional preference in females; however, we may not have been able to detect any preference in males due to their lower sample size.

We think this shift in dispersal direction resulted from the change in density. In the first period, the population size stagnated at a third of what was observed at the end of the study, meaning that there were still many vacant territories in the northern parts of the Hungarian Great Plain, where most imperial eagles reside (Horváth 2022; Zsinka, Pásztory‐Kovács, et al. 2024). However, in the second period, the population increased exponentially. Since we showed that birds from high‐density areas select territories in lower‐density habitats, this population growth could have facilitated the range expansion towards the South as low‐density areas in the North became increasingly scarce. The preference for southern regions over similarly low‐density areas in the West can be explained by the larger contiguous open lowlands in the South, which are the most suitable habitats for the imperial eagle.

### 4.4 High Philopatry Despite High Mobility

Although some individuals have explored areas over 1000 km from their natal site, all birds for which we could determine NDDs have returned to breed in their natal Pannonian population. This population‐level philopatry was previously suggested by GPS telemetry studies for the Pannonian and other resident populations as well (Prommer et al. 2015; Horváth et al. 2018; Korepov 2023; Rymešová et al. 2023; Schmidt et al. 2023).

We must consider the possibility that we failed to detect emigration to other populations in the case of color‐ringed and DNA‐profiled birds. The reason is that the detection or reporting probability of color‐ringed birds might have been lower in some areas outside of the Pannonian Region, where the monitoring of imperial eagles was less intensive, and we only used DNA profiling data from Hungary. However, considering that all of the GPS‐tracked birds (for which we precisely know breeding locations) settled in the Pannonian population and that no bird after the age of 3 cy was recorded in another breeding population, we can conclude that migration to other distant populations is negligible.

Regarding migration between the two subpopulations of the Pannonian population, we detected five movements (~4% of all recorded natal dispersal cases) from the eastern to the western subpopulations. This 4% migration rate is consistent with the only low genetic differentiation of the western and eastern parts found by Vili et al. (2009). The resulting gene flow is beneficial for maintaining genetic diversity and decreasing inbreeding. However, as we have observed only one‐way movement toward the West, further research should give attention to possible source‐sink dynamics between the subpopulations.

Assuming 10.9 km as a median territory diameter (based on Thiessen polygon areas), about 50% of males recruit within four, and 50% of females within six territorial units. We interpret this as high philopatry even on a within‐population scale, considering that birds may scout areas up to a hundred territorial units away from their natal nest during their floater phase.

Such high philopatry, despite high vagility in birds of prey, is not unusual (Ferrer 1993; Literák et al. 2022; García‐Macía et al. 2023; Penttinen et al. 2024; but see for example Limiñana et al. 2012) and has important implications for their conservation. For the imperial eagle, it suggests the genetic uniqueness of the Pannonian and similar isolated populations, while also highlighting their elevated risk of extirpation.

### 4.5 Combined Marking Methods

Our data on NDDs originated from three monitoring methods with different properties. Color ringing provided presence data with no strict spatial truncation but with a low detection rate compared to the other methods. In contrast, DNA profiling provided a higher detection rate and high certainty of breeding status, but at a higher sample cost along with spatial truncation due to our limited study area. GPS tracking had the benefit of a high detection rate with no spatial truncation, but at a much higher cost, resulting in a low sample size, which by itself would not have been sufficient to study natal dispersal patterns.

Most of our NDD data originates from DNA profiling, where spatial truncation occurred, suggesting that our NDD estimates are downwardly biased, especially for the longer‐dispersing females. Excluding the data obtained from DNA profiling, the median NDDs for females and males are 73.1 and 35.9 km, respectively, showing a more pronounced sex bias than the above reported 57.6 and 35.9 km, estimated for all the sampled birds. This implies that we likely underestimated the magnitude of sex bias.

The DNA profiling method of genotype matching chicks and breeding adults has rarely been used in natal dispersal studies (Kylmänen et al. 2023; Penttinen et al. 2024). Our study demonstrates that despite its spatial truncation, it can serve as a valuable, cost‐efficient tool for investigating natal dispersal in raptors on a within‐population spatial scale. We recommend its use for territorial raptors with low population size, for which GPS tracking of individuals in great numbers is not feasible. For species where chicks are already being ringed on a regular basis, obtaining DNA samples from chicks and collecting shed feathers around the nest site can also be incorporated into the ringing schedule with small extra effort.

### 4.6 Conclusion

We demonstrated that natal dispersal in the imperial eagle is characterized by strong philopatry and longer dispersal distances in females and is likely influenced by both intraspecific competition and conspecific attraction.

Our findings suggest that despite its exponential growth, the Pannonian population is unlikely to come into contact with other populations in the near future, given that the nearest ones—in Macedonia and Bulgaria—are approximately 600 km away, about three times the maximum NDD we observed. Therefore, these populations should be considered separate conservation units, and management should aim to facilitate gene flow between them to restore a functional metapopulation structure, for example, by reintroducing intermediate stepping‐stone populations.

Furthermore, although we only detected movements from the eastern to the western subpopulation, implying possible source‐sink dynamics between the two with a risk of biased sex ratios, our sample size in the West was very limited. Therefore, we urge natal dispersal studies on chicks from those regions to obtain more reliable estimates of migration rates.

Our results will contribute to developing more accurate population viability models for the species, further supporting its effective conservation.

## Author Contributions

**Bernadett Zsinka:** conceptualization (equal), data curation (equal), formal analysis (equal), investigation (equal), methodology (equal), visualization (lead), writing – original draft (lead). **Szilvia Kövér:** conceptualization (equal), formal analysis (equal), methodology (equal), supervision (equal), visualization (supporting), writing – original draft (supporting), writing – review and editing (equal). **Márton Horváth:** conceptualization (equal), data curation (equal), funding acquisition (lead), investigation (equal), project administration (equal), resources (equal), supervision (equal), writing – review and editing (supporting). **Nóra Vili:** investigation (equal), writing – review and editing (equal). **Veronika Szabó‐Csonka:** formal analysis (supporting), investigation (supporting). **Krisztián Szabó:** investigation (supporting), writing – review and editing (equal). **Szilvia Pásztory‐Kovács:** conceptualization (equal), data curation (equal), formal analysis (equal), investigation (equal), methodology (equal), project administration (equal), resources (equal), supervision (equal), visualization (supporting), writing – original draft (supporting), writing – review and editing (equal).

## Conflicts of Interest

The authors declare no conflicts of interest.

## Supporting information

File **S1.**

Data S1.

Data S2.

Data S3.

## Data Availability

Data and code needed to reproduce the analyses are provided as Supporting Information. Since the eastern imperial eagle is a sensitive species, nest locations are not shared.
