# Supplementary material for: Sex‐Biased and Density‐Dependent Natal Dispersal in a Highly Mobile but Philopatric Raptor
Source: Ecol Evol. 2025 May 26;15(5):e71487. doi: 10.1002/ece3.71487 (PMC12104666; doi:10.1002/ece3.71487)
Supplement: Supplementary file 1 — File S1. [file ECE3-15-e71487-s003.pdf]

Supporting Information for

„Sex-biased and density-dependent natal dispersal in a highly mobile but philopatric raptor”

Bernadett Zsinka[1,2], Szilvia Kövér[1*], Márton Horváth[3], Nóra Vili[1], Veronika Szabó-Csonka[1], Krisztián Szabó[1], Szilvia Pásztory-Kovács[1]

[1]*Department of Zoology, University of Veterinary Medicine Budapest, Rottenbiller utca 50, H-1077, Budapest, Hungary*

[2]*Lendület Ecosystem Services Research Group, Institute of Ecology and Botany, HUN-REN Centre for Ecological Research, Alkotmány út 2-4, H-2163, Vácrátót, Hungary*

[3]*MME BirdLife Hungary, Költő utca 21, H-1121, Budapest, Hungary*

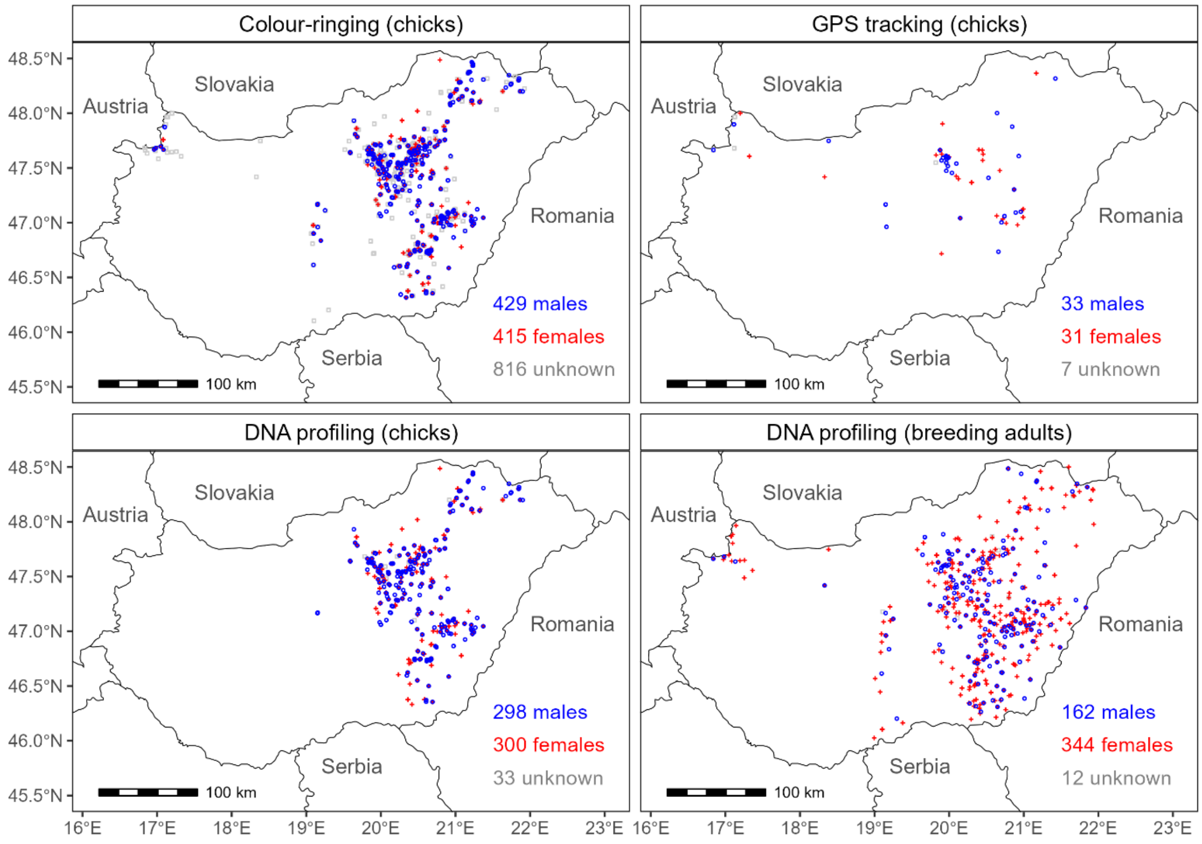

Figure S1. Locality of eastern imperial eagle chicks and breeding adults identified in Hungary between 2011 and 2022. Locality indicates natal nest for chicks and breeding site for breeding adults. Chicks were either marked through colour-ringing, GPS tracking, DNA profiling, or the combination of the above. Unknown sex means that molecular sexing was not carried out or was unsuccessful.

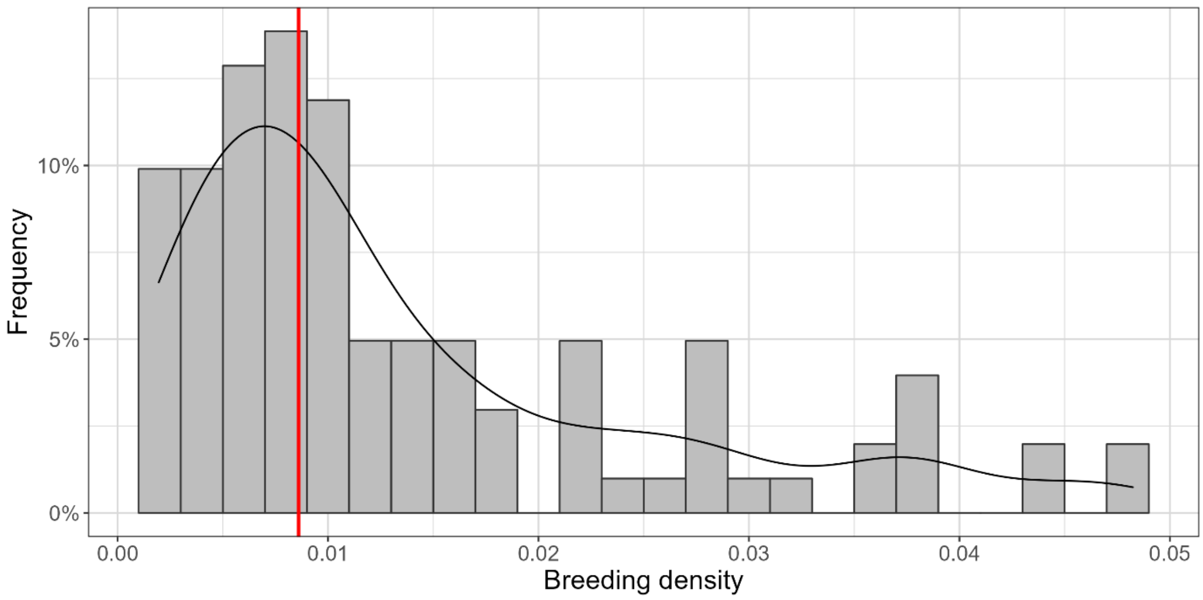

Figure S2. Distribution of breeding site densities. Estimated from our model of *density difference*, the red line represents the natal density value from which birds disperse in equal proportions to lower- and higher-density breeding sites.

Table S1. Estimates of the general linear mixed model investigating the relationship of natal dispersal distance (NDD, log-transformed) with sex ('male' as reference), natal density (log-transformed, scaled) and natal period (two-level factor, '2012–2014' as reference) in 38 male (**outlier male with NDD of 1.8 km excluded**) and 62 female eastern imperial eagles hatched in Hungary between 2012 and 2018. Natal territory ID and natal year (categorical) were set as crossed random intercepts. Effects in bold were significant (p < 0.05).

Response variable: log (NDD)					
Explanatory variables	Estimate	SE	df	t-value	p-value
**Intercept**	**3.395**	**0.135**	**7.978**	**25.104**	**<0.0001**
**sex (female)**	**0.440**	**0.145**	**76.51**	**3.024**	**0.0034**
log (natal density)	-0.016	0.073	67.04	-0.215	0.8302
natal period (2015 – 2018)	0.383	0.160	4.980	2.390	0.0626
Random effects	SD				
natal territory ID	0.336				
natal year	0.093				
Residual	0.605				

Table S2. Estimates of the general linear mixed model investigating the relationship of density difference (log (breeding density) - log (natal density)) with sex ('male' as reference), natal density (log-transformed, scaled), natal dispersal distance (NDD, log-transformed, scaled) and natal period (two-level factor, '2012–2014' as reference) in 38 male (**outlier male with NDD of 1.8 km excluded**) and 62 female eastern imperial eagles hatched in Hungary between 2012 and 2018. Natal territory ID and natal year (categorical) were set as crossed random intercepts. Effects in bold were significant ($p < 0.05$).

Response variable: density difference (log (breeding density) – log (natal density))					
Explanatory variables	Estimate	SE	df	t-value	p-value
**Intercept**	**-0.518**	**0.144**	**89.57**	**-3.609**	**0.0005**
sex (female)	0.062	0.170	92.58	0.366	0.7153
**log (natal density)**	**-0.578**	**0.079**	**71.20**	**-7.289**	**<0.0001**
**log (NDD)**	**-0.253**	**0.092**	**94.59**	**-2.740**	**0.0073**
natal period (2015 – 2018)	-0.116	0.165	95.00	-0.707	0.4810
Random effects	SD				
natal territory ID	0.240				
natal year	0.000				
Residual	0.732				
